# Seminoma of Testis Masquerading as Orchitis in an Adult with Paraplegia: Proposed Measures to Avoid Delay in Diagnosing Testicular Tumours in Spinal Cord Injury Patients

**DOI:** 10.1100/tsw.2008.30

**Published:** 2008-02-06

**Authors:** Subramanian Vaidyanathan, Peter L. Hughes, Paul Mansour, Bakul M. Soni

**Affiliations:** ^1^ Regional Spinal Injuries Centre, District General Hospital, Southport, PR8 6PN, UK; ^2^ Department of Radiology, District General Hospital, Southport, PR8 6PN, UK; ^3^ Department of Cellular Pathology, District General Hospital, Southport, PR8 6PN, UK

**Keywords:** spinal cord injury, testis, orchitis, seminoma

## Abstract

Orchitis is common in adult male spinal cord injury (SCI) patients and, therefore, both health professionals and SCI patients themselves tend to attribute testicular swelling to orchitis, with a consequent potential delay in the diagnosis of testicular tumours. A 37-year-old man with paraplegia developed swelling of the right testis. With a presumptive diagnosis of acute bacterial orchitis, he was prescribed ciprofloxacin while awaiting an ultrasound scan. Ultrasound examination of the testis 4 weeks later showed a moderate hydrocele, enlargement and altered echogenicity of both the epididymis and testis, and features of mass-like lesions within the substance of the testis. As these changes might merely have represented a partly treated infection, a follow-up scan was carried out 2 weeks later, which revealed a lobulated mass of mixed echogenicity within the testis and a focal area of increased echogenicity indicative of calcification. A radical orchidectomy performed 19 days later revealed a seminoma. To prevent delay in the diagnosis of testicular tumours in SCI patients, we propose the following measures: (1) patients who develop swelling of the testis should consult a physician as soon as possible for clinical examination; blind antibiotic therapy should be avoided if possible; (2) if clinical examination reveals a hard swelling of the testis and the typical features of acute urinary infection are absent, an ultrasound scan of the scrotum should be performed as soon as possible; (3) in patients with equivocal ultrasound findings, ultrasound-guided, fine-needle aspiration cytology may allow an early diagnosis of testicular malignancy; (4) education of SCI patients and their caregivers is needed to implement these recommendations.

## BACKGROUND

Adults with spinal cord injury (SCI) are at greater risk of developing epididymo-orchitis than are able-bodied individuals. Researchers from Leeds, U.K., found that 38.5% of SCI patients had suffered at least one episode of epididymo-orchitis[1]. Ku and associates from Seoul, Republic of Korea[2], observed that over a period of

17 years, 39 of 140 male SCI patients (27.9%) were diagnosed with epididymo-orchitis. Given this increased incidence, both health professionals and SCI patients themselves tend to attribute swelling of testis, without preceding trauma, to acute bacterial orchitis. The diagnosis of testicular tumours in this group of patients may therefore be delayed.

National guidelines issued in 2002[3] recommend starting empirical antibiotic therapy in all patients with epididymo-orchitis before microbiology results are available. Where the infection is likely to be caused by enteric organisms, oral ciprofloxacin (either 200 mg twice daily for 14 days or 500 mg twice daily for 10 days) is the antibiotic of choice. If there is no improvement in the patient's condition after 3 days, the diagnosis and treatment should be reassessed; reassessment is also required if swelling and tenderness persist after the course of antibiotics is completed, although in some cases symptoms can take longer than this to settle. Differential diagnoses to consider in these circumstances include abscess formation, mumps epididymo-orchitis, infections caused by mycobacteria or fungi, testicular ischaemia or infarction and, importantly, malignancy. Guidelines on the management of suspected orchitis in people with SCI are not currently available.

We describe an SCI patient with testicular swelling who was initially prescribed ciprofloxacin for a presumptive diagnosis of acute bacterial orchitis, but who was subsequently shown to have a testicular tumour. We discuss possible measures to prevent delay in the diagnosis of testicular tumours in patients with SCI, and we hope that this report will act as a catalyst for the development of guidelines for the investigation and treatment of scrotal swelling in this group of patients.

## CASE REPORT

In 1997, a 28-year-old man sustained T-6 complete paraplegia in a motorcycle accident. Following rehabilitation, he was able to resume full-time employment, managing his bladder by intermittent self-catheterisation. One Friday in June 2007, he noticed swelling of his right testis and on the following Monday, he contacted the spinal injuries unit where he was seen the same day. He had previously developed testicular swelling while playing wheelchair basketball, but on this occasion he had not played basketball for 4 or 5 weeks. On examination, his right testis was found to be enlarged and very hard, while the left testis was small. He did not feel unwell and he was not pyrexial. After taking a sample of urine for microbiology, he was prescribed oral ciprofloxacin (500 mg twice daily) for 1 week and a request for ultrasound examination of the testes was submitted. The urine sample grew coliform bacteria, sensitive to ciprofloxacin. At the end of the first week of antibiotics, the prescription was renewed for a further 10 days by the general practitioner, following which it was changed to oral Co-Fluampicil (combined flucloxacillin 250 mg and ampicillin 250 mg) every 6 h for yet a further week.

Ultrasound examination performed 4 weeks after presentation showed the scrotum and left testis to be normal. On the right side, there was moderate hydrocele, together with enlargement and altered echogenicity in both the epididymis and the testis. The appearance of the testis was reported as slightly atypical, with features of mass-like lesions within the testicular parenchyma, thought possibly to represent partly treated infection (Fig. 1). The radiologist recommended a repeat scan to exclude any underlying malignancy if symptoms persisted. Follow-up ultrasound examination was therefore performed 2 weeks later; this showed persistence of the large right-sided hydrocele, extending up into the inguinal canal, and a lobulated mass of mixed echogenicity within the substance of the testis (Fig. 2). A focal area of increased echogenicity suggesting calcification was also noted (Fig. 3). These appearances were considered highly suggestive of a testicular neoplasm. Blood taken for tumour markers (in accordance with current guidelines, The National Comprehensive Cancer Network Practice Guidelines in Oncology, version 1.2007: Testicular Cancer - Workup of a patient with suspicious testicular mass[4]) showed levels of AFP and beta HCG to be within reference ranges. Radical orchidectomy was performed 19 days later. Gross examination of the resected specimen showed replacement of the testis by a pale, slightly nodular, solid tumour, 60 × 45 × 40 mm, with neither cystic areas nor obvious haemorrhage or necrosis.

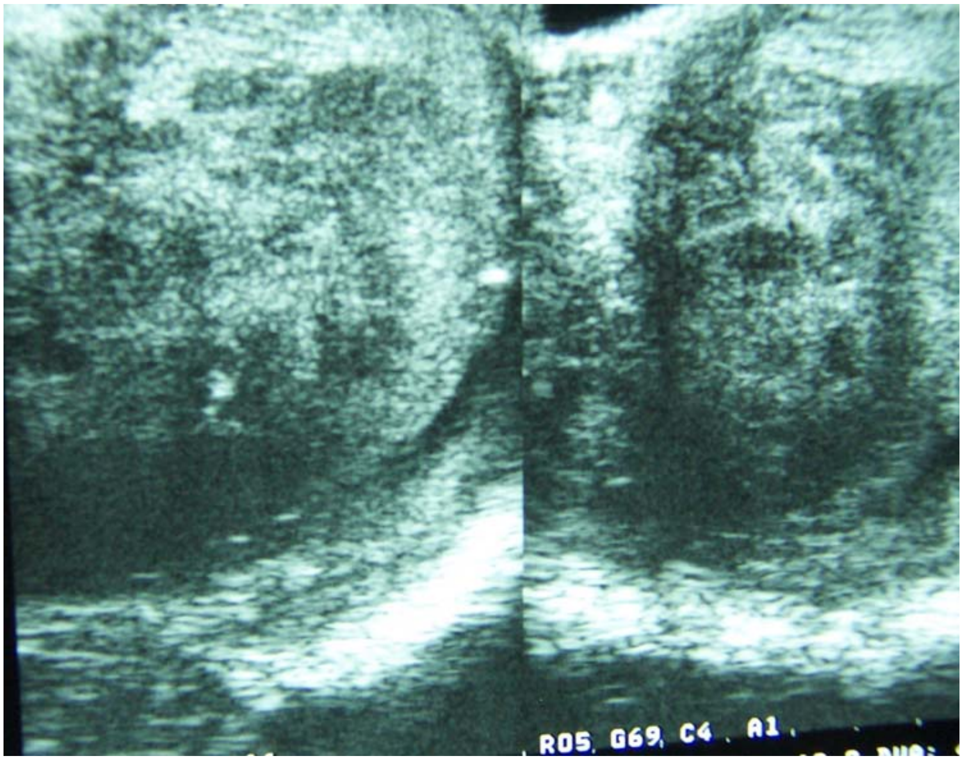

**FIGURE 1.** Ultrasound scan of the right testis, performed on 09 July 2007, shows testicular enlargement with features of mass-like lesions within the substance of testis.

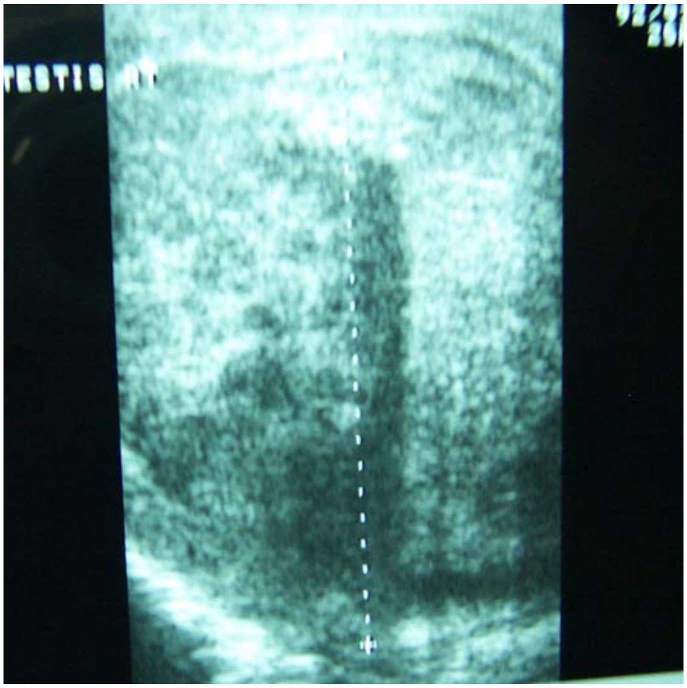

**FIGURE 2**. Repeat ultrasound scan of the right testis, performed on 23 July 2007, shows that a lobulated mass of mixed echogenicity persists within the substance of the testis.

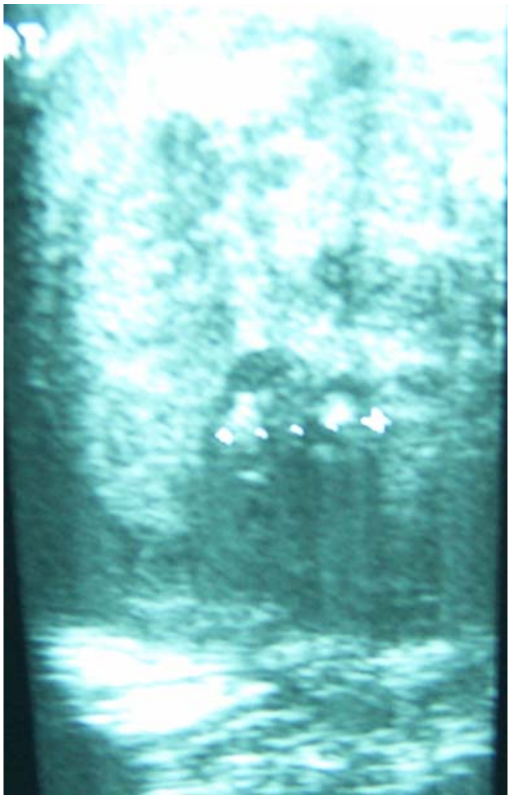

**FIGURE 3**. Focal area of increased echogenicity suggests calcification in the repeat ultrasound scan, performed on 23 July 2007.

Histological examination showed a solid tumour composed of large, round, malignant cells with large nucleoli, intersected by fibrous septae containing a prominent lymphocytic infiltrate, giving a nested appearance to the tumour (Fig. 4). No glandular or papillary structures were present. On immunohistochemical staining, the tumour cells were positive for PLAP and CD117, and negative for pan-cytokeratin (AE1/AE3), CD45, pan-melanoma marker, CD30, AFP, EMA, and vimentin, thus confirming the morphological diagnosis of classical seminoma. Seminoma was invading the spermatic cord, and showed vascular space invasion (pT3).

Computerised tomography of the chest, abdomen, and pelvis showed no evidence of intrathoracic lymphadenopathy or pleural effusion. Under lung setting, no lung parenchymal abnormality was seen. There was no evidence of pulmonary metastases. Liver, pancreas, and kidneys appeared normal. There was no evidence of para-aortic or pelvic lymphadenopathy. The patient subsequently received adjuvant chemotherapy in the form of a single cycle of carboplatin.

## DISCUSSION

It is axiomatic that, for many tumours, early detection improves outcome, often with less intensive treatment. In the U.S., for example, routine surveillance imaging of SCI patients in all Department of Veterans Affairs medical centres allowed the detection of incidental renal tumours at an early stage, so that excision by partial nephrectomy was often feasible[5]. In contrast, the diagnosis of testicular tumours in this group of patients may be delayed because physicians and patients mistakenly attribute testicular swelling to orchitis, as indeed happened in this patient.

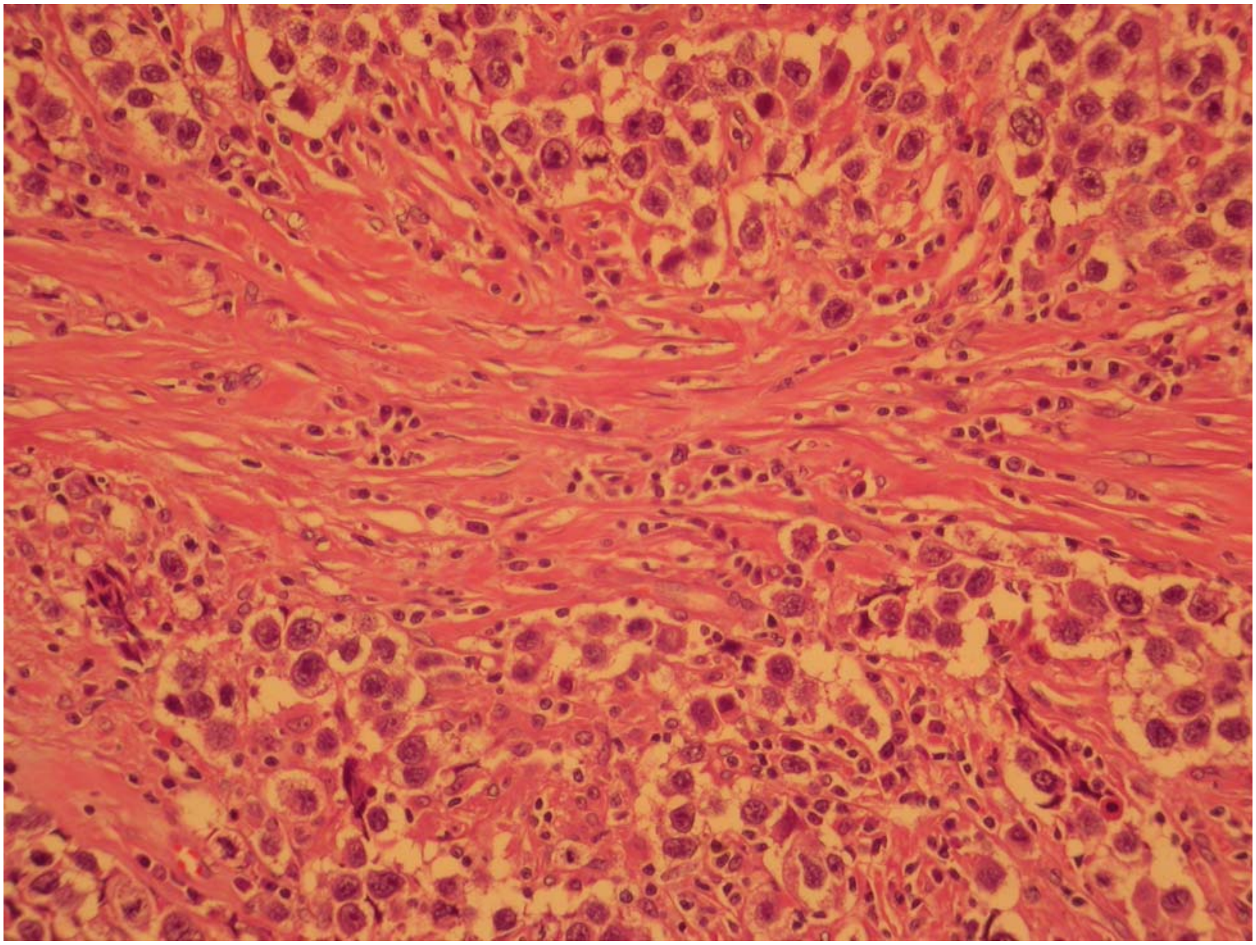

**FIGURE 4.** Histology of right orchidectomy specimen: small nests of classical seminoma cells (top and bottom), intersected horizontally by a fibrous septum (centre). Vascular invasion was present elsewhere.

Clinical history, physical examination, and ultrasound examination provide important clues as to the nature of testicular swellings in general. (Table 1 describes the typical ultrasound findings in orchitis compared with testicular tumours.) In our patient, there was a delay of 4 weeks before testicular ultrasound examination was performed, with further delays of 2 weeks before the follow-up scan and almost 3 more weeks before orchidectomy. Ultrasound-guided fine-needle aspiration cytology (FNAC) of the testicular mass at the time of the first scan would have made the diagnosis of malignancy earlier.

FNAC of the testis with a 23-gauge needle on a 20-ml syringe is a simple, quick, minimally invasive, and painless outpatient procedure[6]. Although interpretation can be difficult in nonspecialist centres, sampling error is less than with biopsy, as several separate punctures can be made and there is no local scarring. It is a reliable, useful, diagnostic tool in the assessment of testicular swellings[7], and can achieve a sensitivity of 95% for seminoma, 88% for Sertoli cell tumours, and 96% for Leydig cell tumours, with a specificity for all three of 100%[8]. Caraway and associates[9] have described in detail the cytomorphologic features of 16 seminomas on FNAC. It also provides suitable material for microbiological examination and helps to avoid unnecessary orchidectomy for benign disease, for example, in distinguishing tubercular epididymitis and epididymo-orchitis from malignancy[10,11]. Needle track seeding has been recognized as a possible, albeit rare, complication of fine-needle aspiration of thyroid cancer[12]. However, there is no evidence to suggest that FNAC of testicular tumours predisposes to local recurrence or inguinal lymph-node metastasis[13].

**TABLE 1**
**Comparison of Testicular Ultrasound Findings in Infection and Seminoma**

| Infection | Seminoma |
|---|---|
| **Epididymitis:** enlargement and hyperaemia of the epididymis, with the arterial waveform changing from its normal triphasic to a low resistance monophasic pattern.<br>**Orchitis:** diffuse testicular enlargement and hypoechogenicity. Focal areas of markedly reduced echogenicity indicate abscess formation. Reactive hydrocoeles are common and may become infected, forming a pyocoele. Severe swelling may compromise the vascular supply, causing secondary focal or diffuse infarction of testis or epididymis. | Nonspecific ultrasound appearances, with single or multiple masses, which are usually hypoechoic relative to normal testis. Cystic change or hyperechoic areas of haemorrhage may be present. Of particular importance is the presence of 2- to 5-mm calcifications, which have been termed macrocalcifications. These may be seen either in the presence or absence of a mass. The presence of macrocalcification should be assumed to indicate a germ cell tumour, prompting testing for tumour markers and a search for metastases. |

An alternative approach to FNAC may be explorative surgery via inguinotomy and preventive clamping of the spermatic cord. The suspicious lesion may be completely removed with biopsy of the resection margins. Nonpalpable lesions may be removed under ultrasonographic guidance. The testicle is only preserved when frozen section reveals a benign lesion and margins are negative[14]. However, in patients with SCI, we tend to follow a very conservative approach even in those clinical situations for which surgery is the standard treatment, as risks of anaesthesia and surgery are greater than in able-bodied individuals. For example, we adopted a nonoperative method of treating extraperitoneal rupture of urinary bladder in a SCI patient successfully[15]. Further, FNAC does not have the same potential for seeding or local spread that needle core biopsy has. Therefore, we recommend the use of FNAC in selected SCI patients with testicular swelling in whom findings of clinical and ultrasonographic examination are equivocal.

In our patient, a second ultrasound scan 6 weeks after presentation suggested focal calcification and it was this finding that raised the alarm (as the presence of macrocalcification on ultrasound examination should be assumed to indicate a germ cell tumour). In other words, even though the patient reported promptly to the spinal unit within 72 h of noticing a swollen testis, we waited for more than 6 weeks until the features of malignancy were obvious on ultrasound scan. A reflection on this case led us to formulate changes to our current clinical practice to facilitate prompt diagnosis of testicular neoplasms in SCI patients (Table 2).

## CONCLUSION

In order to prevent delay in the diagnosis of testicular tumours in SCI patients, we propose the following measures: (1) patients who develop testicular swelling should consult a physician as soon as possible for clinical examination; blind antibiotic therapy should be avoided; (2) if clinical examination reveals a hard swelling and typical clinical features of acute urinary infection are absent, ultrasound scan of scrotum should be performed as soon as possible; (3) in patients with equivocal ultrasound findings, ultrasound-guided, FNAC will facilitate early diagnosis of testicular neoplasms; (4) education of SCI patients and their caregivers is necessary in order to implement these recommendations.

**TABLE 2**
**Summary of Current Clinical Practice in the North West Regional Spinal Injuries Centre, Southport, U.K. and Suggested Measures to Avoid Delay in Diagnosis of Testicular Tumour in Adult SCI Patients**

| Clinical Scenario | Previous Practice | Revised Practice to Prevent Delay in Diagnosis of Testicular Tumour | Rationale for Revised Clinical Practice |
|---|---|---|---|
| A SCI patient who has developed a swelling in scrotum (or his caregiver) telephones the spinal unit and informs a health professional of his condition. | A health professional advises the patient over the telephone to take antibiotics by mouth on the presumption that the patient has developed orchitis. | A physician should speak to the patient and ask following questions:<br>• Does the patient have symptoms of urinary infection such as raised temperature, increased spasms, or general malaise?<br>• Is the swelling confined to scrotum or does it extend to groin?<br>• Did the patient sustain injury to scrotum, e.g., during transfer from toilet seat to wheelchair?<br>The physician should recommend antibiotic therapy only if the patient has definite symptoms of urinary infection. | SCI patients, who develop acute bacterial orchitis, invariably manifest other features of urinary infection at least during the initial phase. Therefore, if a patient notices scrotal swelling in the absence of clinical features of urine infection or scrotal trauma, the spinal cord physician should be wary of making a diagnosis of acute bacterial orchitis and consider other possible diagnosis, including neoplasia. A testicular tumour is intrascrotal while an inguinal hernia extends into groin. |
| | The importance of urgent physical examination appears not to be appreciated by many patients and health professionals alike. Furthermore, there is often a delay before the patient visits the spinal unit for clinical examination. | A thorough clinical examination is mandatory for all such patients and should be carried out within the next two working days. | The European Association of Urology guidelines[16] state that testicular cancer is usually diagnosed by physical examination and generally appears as a painless, unilateral, intrascrotal mass. |
| Investigations for an SCI patient with a scrotal swelling. | Ultrasound scan of scrotum is requested for most patients, but usually on a routine basis (within 2–4 weeks). | Following physical examination, an urgent ultrasound scan of the scrotum should be requested for patients with hard, irregular swelling of the testis, or those in whom distinctive clinical features of urine infection were absent when the swelling occurred. In the remaining patients, a routine ultrasound scan should be carried out preferably within 10 days. | The European Association of Urology guidelines[16] state that ultrasound has an important role in determining whether a mass is intra- or extratesticular. The sensitivity of scrotal ultrasound to detect a testicular tumour is almost 100%.<br>The National Clinical guidelines recommended for use in Scotland - SIGN Publication number 28, September 1998[17] recommend that patients suspected of harbouring a testicular malignancy should undergo an ultrasound scan of both testes. |
| Equivocal findings on ultrasound examination of testis. | Follow-up ultrasound scan is requested and this is carried out usually within a further 2–3 weeks. | Ultrasound-guided FNAC is recommended. | Ultrasound-guided FNAC of testicular lesions is a very reliable technique in predicting malignancy with high sensitivity and specificity. |
